# A scoping review of HIV epidemiologic, sociocultural and programmatic studies related to transgender women and men who have sex with men in Cambodia, 1999-2019

**DOI:** 10.1371/journal.pone.0254490

**Published:** 2021-07-16

**Authors:** Jan W. de Lind van Wijngaarden, Frits van Griensven, Ly Penh Sun, Stephen Wignall

**Affiliations:** 1 Independent Consultant, Chonburi, Thailand; 2 Institute of Health Research and Innovation, Bangkok, Thailand; 3 Department of Epidemiology and Biostatistics, University of California at San Francisco, San Francisco, California, United States of America; 4 National Centre for HIV/AIDS, Dermatology and STD, Phnom Penh, Cambodia; 5 FHI360/LINKAGES, Phnom Penh, Cambodia; International AIDS Vaccine Initiative, UNITED STATES

## Abstract

**Background:**

Cambodia is widely credited for its successful HIV epidemic control. However, in recent years there have been signs of increasing HIV prevalence among men who have sex with men (MSM) and transgender women (TGW). This paper reviews HIV epidemiological, social science and HIV program implementation studies conducted over the past 20 years to explore possible reasons for the rising HIV prevalence among these groups and to formulate recommendations for improved policies, HIV programmatic interventions and further research.

**Methods:**

For this scoping review, we searched the PubMed and Google Scholar databases for scientific publications related to HIV and MSM and TGW in Cambodia published since 1999. From each of the returned citations we subsequently studied reference lists to find additional data sources. We also searched websites for reports commissioned by national and international governmental and non-governmental organizations.

**Results:**

Twenty-seven relevant studies and papers were found and reviewed; most were epidemiological in nature. Recent epidemiological studies and reports show an increase in HIV prevalence among Cambodian MSM and TGW. The epidemiology of HIV infection in these groups has been relatively well-described and analyzed. While initially MSM and TGW were grouped together, in more recent years they have been studied in their own right, recognizing their specific HIV and other prevention needs. Few studies were found investigating Cambodian same-sex cultures and social and cultural contexts in which HIV transmission among MSM and TGW occurs. A few evaluation studies were found, but it remains unknown how effective current HIV service implementation modalities are, or how successful strategies to increase access to essential HIV prevention, testing and treatment services have been employed for MSM and TGW in Cambodia.

**Conclusions:**

Research about Cambodian MSM and TGW in the context of HIV primarily concerns bio-behavioral knowledge generation. Cambodia is unlikely to achieve control of the HIV epidemic among MSM and TGW without doing better in-depth social science research on its multiple sexual- and gender minority cultures, and without understanding what differentiated implementation modalities, strategies and approaches are most effective to address HIV among its increasingly diverse MSM and TGW populations.

## Introduction

Men who have sex with men (MSM) and transgender women (TGW) have been disproportionally affected by human immunodeficiency virus (HIV) infection in every part of the world [1–3]. Cambodia has been successful in controlling the spread of HIV in its general population [4, 5], which was initially mainly driven by female sex work [6, 7]. A recent analysis noted that overall HIV incidence has decreased and antiretroviral treatment access and coverage has increased in the past 20 years [8]. However, national HIV prevalence among MSM has risen from 2.1% in 2010 to 4.0% in 2019, and among TGW it more than doubled from 4.2% in 2012 to 9.6% in 2019 [9]. Interventions for MSM and TGW have struggled to increase levels of condom use and HIV testing uptake among their target populations [10]. Despite earlier achievements to control the HIV epidemic, there is now an urgent need to refocus responses to key populations at higher risk by expanding their access to community and facility-based HIV testing and retention in HIV prevention, treatment and care services [8].

This scoping review paper brings together literature on HIV epidemiology among MSM and TGW and related social science and implementation studies conducted in Cambodia between 1999 and 2019. The goal is to identify reasons for the rising HIV prevalence among these groups and to derive recommendations for improved policies, HIV programmatic interventions and further research.

## Methods

No specific protocol was developed for this scoping review, but we followed the PRISMA scoping review checklist [S1 Checklist]. We searched the PubMed [11] and Google Scholar [12] databases for scientific publications between 1999 and 2019, using the following medical subject heading terms: AIDS OR HIV AND (TG OR TGW OR transgender OR transgender women OR MSM OR LGBT OR men who have sex with men OR homosexual AND Cambodia). We also searched the AIDS Data Hub [13] for relevant reports commissioned by national and international governmental and non-governmental organizations. Finally, we cross-checked reference lists of included documents to find additional data sources.

## Results

**Fig 1:**
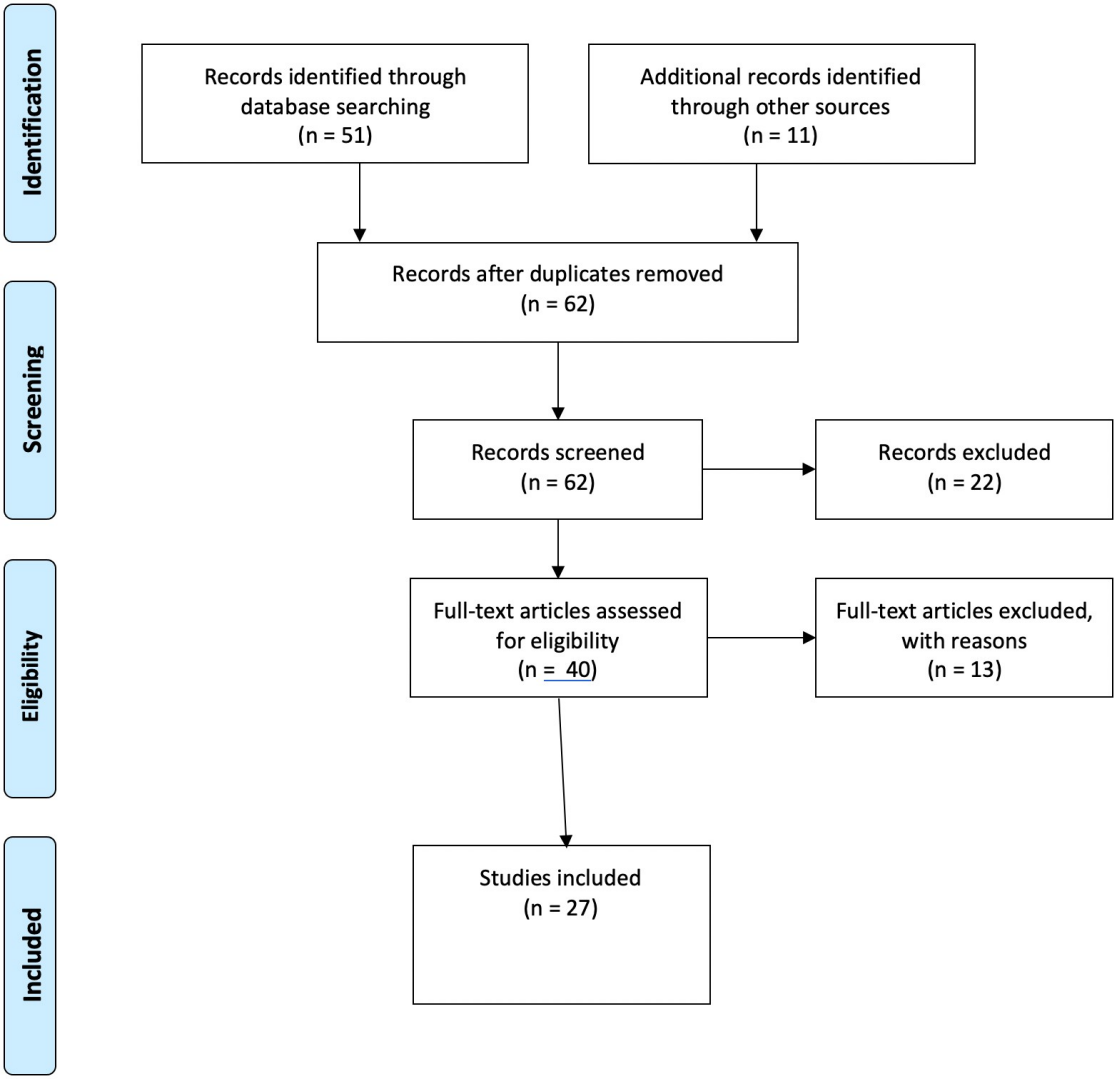
PRISMA flow diagram of identified, screened, eligible and included publications.

The literature search resulted in 51 papers, of which 29 were about Cambodia and 22 had a regional or global focus or were about other countries. Of the 29 papers about Cambodia, 16 concerned MSM and/or TGW. Further searches identified an additional 11 papers and reports about MSM and TGW available in the public domain, hence a total of 27 papers and reports were included in this scoping review (Fig 1). Twelve papers and reports were only about MSM, nine were only about TGW and six were about both MSM and TGW.

### Epidemiologic and related socio-behavioral studies on men who have sex with men and transgender women

#### Men who have sex with men

The HIV epidemic among MSM in Cambodia was first detected in 1999–2000 and published in 2004. It found an HIV prevalence of 14.4% and syphilis prevalence of 5.5% in a sample of 206 MSM (including 39 TGW) based in Phnom Penh [14] (Table 1). Only a minority (22.9%) of the sample self-identified as ‘homosexual’ and 61.0% reported sex with both men and women. The majority of participants (82.6%) were engaged in sex work. A third of the sample reported sex with a female sex worker in the past 6 months. Consistent condom use with male partners in the past month was 51.6%; with female partners it was 74.7%. The authors concluded that “complex sexual networks indicate that [MSM] act as a bridge between higher and lower HIV prevalence populations” and called for “better prevention efforts structured around behaviors rather than sexual identities” [14].

**Table 1:** HIV prevalence among populations of men who have sex with men and transgender women in Cambodia, 1999–2019.

Men who have sex with men and transgender women				
Year	HIV prevalence (%)	Number enrolled (N)	Location	Reference
1999–2000	14.4	206	Phnom Penh	14
2005–2006	5.0	299	Phnom Penh	15
	0.0	249	Battambang and Siem Reap*	
Men who have sex with men and women				
2010	2.2	592	Phnom Penh, Sisophon, Poipet, Battambang, Kandal, Siem Reap, Kampong Cham, Sihanoukville*	16
Men who have sex with men				
2010	2.1	434	Phnom Penh, Sisophon, Poipet, Battambang, Kandal, Siem Reap, Kampong Cham, Sihanoukville*	16
2014	2.3	838	Twelve provinces, of which:	20, 21
	3.0	NA	Phnom Penh	
	5.9	NA	Siem Reap	
	2.5	NA	Bantey Meanchey (Poipet)	
	1.7	NA	Sihanoukville	
	0.7	NA	Kompong Cham	
	0.5	NA	Kendal	
	0.5	NA	Battambang	
2019	4.0	1,569	Twelve provinces, of which:	9
	6.9	336	Siem Reap	
	6.1	213	Phnom Penh	
	4.1	266	Battambang	
	3.7	269	Banteay Meanchey	
Transgender women				
2005–2006	9.2	197	Three provinces, of which:	15
	17.0	102	Phnom Penh	
	2.0	95	Battambang and Siem Reap	
2014–2015	4.2	891	Eight urban centers, of which:	23
	5.8	313	Phnom Penh	
	8.8	114	Siem Reap	
	2.0	464	Other locations combined	
2016	5.9	1,375	Phnom Penh and 12 provinces, of which:	23–26
	11.7	111	Banteay Meanchey	
	11.3	124	Siem Reap	
	6.5	541	Phnom Penh	
	5.3	151	Battambang	
	4.2	131	Kendal	
2019	9.6	1,025	12 provinces, of which:	9
	17.7	113	Banteay Manchey	
	16.4	128	Siem Reap	
	14.0	221	Phnom Penh	

In 2005–6, the National Center for HIV/AIDS, Dermatology and STDs (NCHADS) started to include MSM and TGW in integrated behavioral and biological surveillance (IBBS). The 2005 survey sampled 548 MSM and TGW from Phnom Penh (n = 299) and two provinces Battambang and Siem Reap [n = 249]. It was reported that more than one-third of the sample identified as women/TGW (34% in Phnom Penh and 38% in Battambang and Siem Reap). MSM and TGW were not disaggregated for separate analysis in the report, except for HIV prevalence. A large majority of the sample, 65% in Phnom Penh and 82% in the other cities, were under 25 years. Almost half of the sample also reported sex with women in the past month and 22% (Phnom Penh) and 29% in the provinces reported sex with female sex workers. Consistent condom use with male partners was low in the provinces (19–23%, depending on partner type, compared to 49–66% in Phnom Penh). Only 21% of the sample had tested for HIV in the past year; 75% of the sample in the provinces and 68% of those based in Phnom Penh had never tested for HIV. Overall, HIV prevalence was 2.6% among MSM. In Phnom Penh, HIV prevalence was 5% among MSM; in Siem Reap and Battambang HIV prevalence was 0% (Table 1). Sexually transmitted infections (STI) were common; 5% of MSM had a bacterial STI [15].

In 2010, a survey was conducted among 3007 Cambodian men frequenting ‘hot spots’ (defined as entertainment and/or sex work sites or cruising sites) in eight cities. Of the sampled group of 3007 men, 1026 reported to have had sex with men, of whom 434 exclusively with men and the rest with both men and women; the rest (1981) were exclusively heterosexual. HIV prevalence was 2.2% in men who had sex with both men and women (MSMW), 2.1% in men who had sex with men only (MSMO) (Table 1), and 1.6% in men who had sex with women only. Around three-quarters of MSMW and MSMO had had an HIV test in the past 12 months. Interestingly, MSMO and MSMW were more likely to have been reached by outreach workers than men who had sex with women only, but their HIV knowledge was less. The authors suggested that there may be a problem of quality with outreach efforts. Around 11.5% of all MSM (118 out of 1026) reported using crystal methamphetamine (ice) to increase sexual pleasure (also known as ‘chem sex’) and 8.8% (90 out of 1026) used amphetamines (also known as *ya ma* or *ya ba*). Of MSMO 15.2% gave as a reason for not using condoms with a male paid partner (n = 46) that they were ‘too high’ at the time of the most recent sex. The study concluded that more attention needs to be paid to MSMW [16].

In 2014, a cross-sectional survey was conducted among 367 MSM sampled by probability proportion-to size-sampling from villages in Battambang and Siem Reap provinces to assess factors associated with inconsistent condom use [17] and with recent HIV testing [18]. The mean age of the sample was 23.9 years and 62.3% of respondents reported that they always used condoms during sexual intercourse over the past three months, with condom use being highest in the context of sex work and lowest with steady boyfriends or girlfriends [17]. Of the sample, 4.4% used ‘illicit drugs’ in the past three months; these men were significantly less likely to report always condom use than the group who did not (25.0% versus 61.5%). A large majority (83.6%) had been tested for HIV infection at least once, and 65.1% had been tested in the past six months; the majority received the result (98.2%) and counselling (95.7%) for the most recent testing [18]. Those who received some form of HIV education were significantly more likely to have been tested than those who had not. People with a recent STI infection and who perceived that they were at higher risk for HIV than the general population were also more likely to have been tested [18].

In a sub-study focusing on mental health in the same sample of 367 MSM from Battambang and Siem Reap provinces in 2014, 10.7% of men reported suicidal thoughts and 6.7% had attempted suicide in the past three months; 38.8% were classified by the authors as being in poor mental health. Higher levels of psychological distress were independently associated with older age, alcohol and drug use, poor self-reported quality of life, and more condom-less intercourse. The authors suggest that mental health interventions should be integrated with HIV prevention among MSM, also addressing alcohol and other substance use. They call for clinical and non-clinical HIV service providers to screen for mental health symptoms and improve social support for MSM in psychological distress [19].

In 2014, 838 MSM were sampled for a population size estimation and to measure HIV-related risk behaviors and prevalence [20, 21]. The study concluded that there were 31,000 MSM in Cambodia as of 2014, of whom 20,000 were considered ‘reachable’. HIV prevalence in the sample was 2.3%; only 0.6% of MSM under the age of 24 were found to be HIV-infected, versus 4.6% of those aged 25 and above. Those with less education had higher HIV prevalence. Among the 12 provinces/study sites, HIV prevalence was highest in Siem Reap (5.9%) and Phnom Penh (3.0%) (Table 1). Consistent condom use in the past month was 69.4%; 71.6% had been reached by HIV outreach workers, and 66.6% had been tested for HIV in the past six months. The report called for evaluations of current interventions for MSM to establish their effectiveness [20, 21].

In 2019, 1,569 MSM were sampled across 12 provinces as part of a focused IBBS study. Overall, HIV prevalence was found to be 4.0%. Siem Reap had the highest HIV prevalence among MSM at 6.9%, followed by Phnom Penh (6.1%), Battambang (4.1%) and Banteay Meanchey (3.7%). The prevalence of STI ranged from 7.8% and 21.1% across provinces. Consistent condom use by MSM with steady, casual, paid, and paying partners in the past six months was low at 45.6%, 51.5%, 50.3% and 49.7%, respectively. The study found that 15.5% of MSM had used amphetamine-type substances in the past 12 months (an increase compared to the 11.5% found in 2010). Use of online dating apps was associated with higher HIV prevalence (8.7% versus 2.7%). Higher levels of education were linked to higher HIV prevalence (1.7% among those with no formal education compared to 8.7% among those with a university degree) [9].

#### Transgender women

In the above-mentioned 2005–2006 IBBS study, 197 TGW were enrolled, 102 from Phnom Penh and 95 from two provinces (Battambang and Siem Reap). HIV prevalence among TGW overall was found to be 9.2%; in Phnom Penh, it was 17% and in the provinces 2%. Over a fifth of TG (21%) had a bacterial STI (no decimals were provided in the original report) [15].

A study on violence and exposure to HIV was conducted among nearly 1,000 female and TGW sex workers in Phnom Penh in 2006; 140 of the sampled sex workers were TGW. Thirty percent of TGW sex workers reported police brutality during the past 12 months; 41% said they had their money stolen by police; 24% answered they had been raped by a single policeman and 18.2% reported gang-rape by police in the past 12 months. “Gangsters” (i.e. groups of criminally inclined men) were another big problem they faced: 57.6% said to have experienced physical violence by gangsters in the past 12 months; 66.7% reported to have had their money taken, 51.5% answered they had been raped by a such a person and 52.3% reported gang-rape in the past 12 months. In terms of reported violence by clients, 38.3% answered to have been beaten; 18.2% reported robbery; 48.5% said they had been raped by individual clients and 37.5% reported client gang-rape in the past 12 months [22].

In 2014–2015, an IBBS study was conducted among 891 TGW from six urban centers using respondent-driven sampling. Their median age was 23, 90.5% were employed, 80.5% had secondary education or higher. HIV prevalence was 4.2%. Consistent condom use was low (20.3%), especially with paid male sex partners. Fifty-four percent reported having experienced discrimination related to their transgender identity in their lifetime, and 30.3% reported having been raped and/or physically assaulted in the past 12 months. Multivariate analysis revealed that older age, lower education, residing in Siem Reap, receiving payment at first sex, having sex while using drugs, inconsistent condom use during latest anal sex, and low self-esteem were independently associated with HIV infection [23].

In early 2016, another IBBS survey was conducted among 1,375 TGW in Phnom Penh and 12 provincial sites [23–26]. Overall HIV prevalence was found to be 5.9%, with Banteay Meanchey (11.7%) and Siem Reap (11.3%) having the highest prevalence, trailed by Phnom Penh (6.5%), Battambang (5.3%) and Kendal (4.2%); the other sites had sample sizes of <100 and are not presented here. Nearly two thirds (61.9%) did not use a condom during last anal sex; 19.6% had never been tested for HIV, whereas 44.3% had been tested in the past 6 months. Approximately half (470 of 873, 53.8%) of participants reported drinking alcohol in the past 3 months, and 13.5% (118 of 873) reported drinking daily. Forty-five percent used hormone treatments, 39.2% had been sexually abused and nearly a quarter reported job-related discrimination. A further 21.6% had ‘ever’ used drugs, and 7.2% had used crystal methamphetamine in the past 3 months [23]. The authors suggested that TGW should be seen as the highest-risk group for HIV in Cambodia, and suggested HIV testing services should be promoted using language that is acceptable to prospective TGW clients. Self-testing was also suggested as a suitable option to increase HIV testing uptake for TGW [23–26].

A sub-set of the IBBS data collected in 2016 was used for an analysis of condom use with non-commercial partners of TGW. It was found that TGW with more than 10 years of education, living in urban settings, and who perceived they were HIV-infected were more likely to use condoms with non-commercial partners, among other factors [27]. Another paper focused on exposure to gender-based violence and prevalence of symptoms of depression among TGW. Forty-five percent of the sample had symptoms of depression and 21.8% of severe depression. Depression was associated with feelings that coworkers or classmates were not supportive, having difficulties finding employment, having been denied or expelled from housing, facing barriers in accessing health care, having been physically abused or fearing arrest by police. A history of sexual abuse during childhood was also related to experience of depressive symptoms [28].

In 2018, a study was published on the vulnerabilities of TGW involved in sex work. The study notes feelings of self-blame, shame, guilt and “a perceived fatalism observed within the social identity” of TGW; 74% had experienced sexual harassment, 40% cited physical assault in the past 12 months, and 55% said they had been forced to have sex against their will. Several forms of discrimination were reported, including loss of employment (39%) and loss or denial of housing (20%), and to a lesser extent denial of health services (10%) and education opportunities (12%). The police were regarded as a main culprit in stigma and discrimination as well as physical assault [29].

In 2019, 1025 TGW were enrolled as part of IBBS across 12 provinces. Overall, HIV prevalence among TGW was 9.6%. The highest HIV prevalence was in Banteay Meanchey at 17.7%, followed by Siem Reap (16.4%) and Phnom Penh (14.0%). The prevalence of STI among TGW ranged between 13.4% and 57.7% across provinces. Consistent condom use in the past six months with steady, casual, paid, and paying partners was low at 39.1%, 51.2%, 56.1%, and 56.3%, respectively. Whereas for MSM it had been observed that higher levels of education were linked to higher HIV prevalence, for TGW lower levels of education were associated with higher HIV prevalence [9].

### Qualitative studies and assessments on Cambodian sexual- and gender minority cultures

When the first HIV prevalence study was conducted among MSM in 1999–2004, homosexuality was still a sensitive issue in Cambodia and little was known about the lives of MSM and TGW [14). To fill this gap in knowledge, a number of qualitative rapid assessments were conducted between 2002 and 2008 [22, 30–34].

The first of these reports (2003) [30] found “blurred distinctions between appearance, identity and sexual practice”, which the authors claimed would, “mixed with the low provision of information, education and support services, and the relatively high HIV prevalence in the country, [lead] to a high risk of transmitting HIV”. MSM had limited knowledge about HIV infection, low risk awareness, low condom use and significant misconceptions about HIV. The report uncovered a high demand for MSM-friendly counseling and testing services and recommended that targeted HIV outreach programs be set up, taking the diversity of the MSM population into consideration. The authors reported that the term ‘MSM’ was starting to be used as a label to self-describe a homosexual orientation or identity. The authors concluded that the MSM population was ‘complex’ but they distinguished two groups: the *srey sros* (‘charming girls’) or *sok veng* (‘long hair’), referring to what we now would label as TGW, and *pros saat* (‘beautiful men’) or *sok khlei* (‘short hair’), referring to MSM. Another group identified were sex workers who could be part of either of these two categories. They called for more research on “knowledge, attitudes, behavior and practices of MSM, not only in the context of HIV but also in terms of their roles within society” [30].

A second study (2004) showed that MSM had difficulty accessing HIV and other health services due to stigma by health care providers, “particularly MSM classified as long hair” or what we now define as TGW. “MSM short hair” would conceal the fact that they have sex with men when accessing health services. The study suggested that there is an over-reliance on peer-outreach and that other health promotion strategies must also be considered [31].

A discussion paper in 2006 about terminologies introduced by Western HIV and other development organizations in Cambodia elaborated on the conceptual weakness of the term ‘men who have sex with men’, especially since many people covered by this term do not in reality regard themselves, and/or may not regard the social gender of their sexual partners, as ‘men’. The author calls for a better understanding of local meanings of gender, but at the same time acknowledges that some of the concepts introduced by outside organizations, such as empowerment and human rights, can be and have been useful to improve the life of marginalized populations [32].

In 2008, UNESCO in Cambodia published an anthropological study to shed light on same-sex cultures of men in Cambodia, using a life history methodology [33]. It extensively discusses Khmer terms related to gender and sexuality, of which there are only a few. According to the author, the word *ktoey* is a Khmer term for a biological male with feminine characteristics or someone who is sexually deficient or born with incomplete or mixed genitals. Currently, this wording is considered derogatory. There are linguistic terms for men (*pros*) and women (*srey*). There are no words depicting sexual identities or orientations—this was also found in the first modern study of gender and sexuality conducted in Cambodia in the 1990s, which found that most Cambodians “do not consciously reflect on their sexual identity, but rather on themselves as females and males living in Cambodian society” [34]. Similar to the sexual/gender cultures of Thailand and Laos, the sexual identity of a person is not based on their sexual behavior but on their personality traits or ‘character’ (*charek*), traits which are believed to be fundamental and innate. Indeed, many informants in the study [33] mention how young men often engage in sexual relationships with each other or with *ktoey* without having consequences for their masculine self-identity. Stereotypical masculine characteristics can be either ‘firm and tough’ (*reng peng*) or ‘gentle and docile’ (*tuon phluon*); the informants of the study saw men as being born as either one or the other. *Ktoey* are therefore *tuon phluon* and attracted to men who are *reng peng*. *Ktoey* express their identity in two distinct ways: most participants in the study “do not feel it is necessary to express this identity by visible signs. This allows them to blend in with the rest of the population”. However, the second position “involves putting aside the biological sex and assuming the social gender through feminization of one’s personal appearance”. The study criticized the use of the words ‘long hair’ and ‘short hair’ to describe categories of Cambodian MSM, failing to find any understanding of these terms among its research subjects, nor for the term *srey sros* (‘charming girl’), which reportedly were “invented by AIDS organizations”: “no one knows how [these terms] came about and they do not seem to correspond to any reality. […] Hair length is not a reliable variable [for a person’s identity] in as much as a hairstyle can be changed from one minute to the next with a pair of scissors”. It also criticized the term *srey sros*, which was coined by HIV and LGBT rights organizations to replace the derogative word *ktoey*. Some older informants mentioned a hitherto unknown term–*srey soth*–that existed in the past to denote men with feminine characteristics, but without the negative connotation of the word *ktoey*. The study found that relationships between *ktoey* and men often have ‘a notion of hunting, capture and consumption’, where the man behaves in a passive way and expects to be pleasured and taken care of. Whereas relationships can work out well in the short term, in the long term there is often an element of disappointment in relationships between *ktoey* and men, with the man eventually choosing to get married to a woman in order to fulfill his need to have offspring. The same desire also led some *ktoey* in the study to eventually get married as well. Some *ktoey* choose other *ktoey* for a relationship—“carefully pick[ing] out ones whose feminine character is less pronounced than [their] own” [33].

In 2012, a study on social exclusion of lesbians, gay, bisexual and transgender (LGBT) persons in families and communities was conducted among 149 respondents, mostly from Phnom Penh. The study showed that LGBT persons experience high levels of stigma, discrimination, and exclusion in a variety of settings: the home, school, the workplace, health facilities and public spaces. Experiences ranged from being ignored to not being allowed to express oneself or not being recognized in community activities and processes to being subject to domestic and gender-based violence. The authors note that the traditional safety net—the family—“becomes an oppressor so LGBT turn to friends more often for support during critical shocks”. TGW experienced more discrimination compared to lesbians and gays and reported higher rates of exclusion and police harassment, arbitrary arrest and “association with having HIV.” The authors recommended research on the situation and needs of young LGBT (12–18 years old) [35].

In 2016, the state of interventions and rights for LGBT and other populations in Cambodia was reviewed. The authors found that because of a general focus on ‘disease and deviance’ underpinning research interest in these populations, issues other than HIV and health have been overlooked despite their relevance for LGBT communities. These issues include gender-based violence, access to gender-affirmation surgery and hormone therapy for transgender people and a ‘lack of attention for the mental health-, housing-, employment- and other social/economic/emotional needs of non-heteronormative people’ [36]. The same authors also note the increasing level of community-organizing among LGBT groups in an attempt for better human rights [37].

In the same year a study was conducted to determine barriers to HIV and other health services for Cambodian key populations, including MSM and TGW. Data was also collected from representatives of the police force. It found that key populations’ fear of accessing harm reduction and health services and police’s negative attitudes and practices towards key populations present major barriers to HIV prevention efforts in Cambodia. The authors concluded that in order to create an enabling environment and ensure police are allies in the Cambodian HIV response, interventions should tackle underlying negative attitudes among police towards key populations and vice versa [38].

### Intervention studies

A limited number of studies were found that aimed to describe or evaluate HIV programmatic interventions for MSM and/or TGW. A 2013 study described a mobile photo booth and Facebook promotional effort to promote positive health messages among MSM in Cambodia. Fifteen thousand people were directly or indirectly exposed to the a Facebook page promoting male sexual health called ‘MStyle’, “including a significant number of hidden MSM”, according to the author [39].

An evaluation of the Sustainable Action against HIV and AIDS in Communities program among key populations between 2010 and 2015 was published in 2016. Outcome indicators were compared with baseline data for MSM, but no significant change was found in condom and lubricant use in all types of relationships as a result of the project. Regarding STIs, 28.1% of MSM at midterm reported having at least one STI symptom in the past three months compared to 6.1% at the end of the study; out of the group of MSM with at least one STI symptom, 14.1% sought treatment at midpoint, compared to 20.7% at the final assessment. The proportion of MSM who reported having been tested for HIV in the past six months decreased significantly from 94.1% to 77.1%. Of MSM 12.2% reported using illicit drugs at baseline in 2012 in two provincial sites, which dropped to 5.1% in 2014. The authors conclude that the intervention was effective in improving some sexual behaviors and health outcomes among MSM. However, it could not increase condom use and HIV testing rates among this key population [10].

Another study describes a Risk Tracing Snowball Approach (RTSA) intervention aimed at increasing detection of HIV infection in Phnom Penh, focusing mainly on MSM and TG. The rate of newly identified HIV positive cases was significantly lower among RTSA clients than that of walk-in clients during the same time frame (1.8% vs. 3.2%). However, because RTSA clients were significantly less likely to have ever been previously tested for HIV (58.9% vs. 72.7%) and more likely to have had STI symptoms since last HIV testing or lifetime if never been tested for HIV (29.5% vs. 55.2%), the authors conclude that the inclusion of RTSA in addition to walk-in clients can boost HIV case detection [40].

A qualitative study among MSM, TGW and female entertainment workers assessed demand and acceptability for HIV self-testing (HIVST). Almost all participants among the three groups had not heard about HIVST, but all of them expressed high willingness to try it. They perceived HIVST as confidential, convenient, time-saving, and high-tech. Barriers to obtaining HIVST included cost, access, administration technique, embarrassment, and fear of pain. The majority preferred counseling before and after testing [41].

Access to community-based HIV programs was assessed based on an existing sample of 1,375 TGW included in IBBS in 2019. TGW who were reached by HIV programs were significantly more likely to report using condoms consistently with paying partners, but less likely to report using them consistently with non-commercial male partners. Less than half of TGW in the national survey had access to HIV services. The authors conclude that innovative strategies and culturally sensitive interventions should be put in place to reach and respond to the needs of sub-groups of TGW who are less likely to be reached by the existing venue-based outreach approaches [42].

## Discussion

Most of the literature on MSM and TGW in Cambodia has focused on HIV/STI prevalence and HIV-related knowledge and risk behaviors. It showed a continuing high burden of HIV and risk for HIV acquisition among MSM and TGW since 2010. Consistent condom use among MSM has not increased according to the 2019 IBBS survey [9]; it was found to be around 50%, similar to the level recorded in the very first prevalence survey conducted in 1999 [14]. On the positive side, whereas a 1999 study found that slightly less than 20% of MSM had ever tested for HIV, the latest data show that 51.5% of MSM and 64.4% of TGW had been tested in the past 12 months [9], an impressive improvement.

Several studies were found focusing on exploring social and cultural contexts in which HIV epidemics among MSM and TGW occur. One study explored roots of homosexuality and transgender identities from the perspective of the Khmer language, history and culture [33]. Poorly conducted social research can do more harm than good. For example, the labeling of MSM by the authors of a 2003 assessment [30] led to a decade of dividing same-sex attracted and gender-nonconforming Cambodians into two groups: ‘long hair’ MSM and ‘short hair’ MSM. This created a false sense that there was a clear and agreed understanding of Cambodian notions of homosexuality and transgender culture(s) on which HIV practitioners and policy makers could base their HIV service programs. Initially TGW were made invisible and lumped under the same label as MSM; only in 2013, NCHADS adopted new Standard Operating Procedures for HIV programming, in which the long hair/short hair MSM misrepresentation was resolved and TGW were identified as one of four key populations, distinct from MSM [43].

The results of our scoping review suggest that even in 2020, there remains a lack of research studies describing modern, urban same-sex cultures of (especially young) MSM and TGW in Cambodia. However, this conclusion should be made with caution, as important social science studies may not have been captured by the search methods used.

The 2019 IBBS, for the first time, focused on the use of online dating apps and its role in HIV transmission [9]; however, online sexual cultures (especially important for young MSM and TGW) and how they can be harnessed for HIV prevention and increased HIV testing uptake remain understudied in Cambodia.

Whereas bathhouses have played a major role in HIV epidemics among MSM in major urban areas in the world [44–46], no qualitative studies on the sauna and spa scene in Cambodia were found; such studies, once conducted, could help inform and design effective HIV prevention and testing interventions for those who are visiting these locations.

Several research studies reviewed in this paper pointed out that MSM and TGW face high levels of stigma and discrimination in Cambodian society, and two studies discovered high levels of depression and poor mental health among MSM [19] and TGW [29]. This was often linked to ‘adverse childhood events’ and strained relationships with family, colleagues and other significant people [19, 26]. Further data is needed to investigate how MSM and TGW (learn to) manage their sexual/gender identity vis a vis family members, how they secure support of friends or community members, or maintain relationship with neighbors or colleagues [33] (see [47] for an example on this from Thailand). Additional social science studies may improve our understanding of these issues which in turn can help strengthen strategies for improved psychosocial support, including counseling [47]. This, of course, is conditional on the availability of such services for MSM and TGW; currently mental health services barely exist in Cambodia [19, 48].

HIV transmission and prevention cannot be seen in isolation from a range of related health and social issues, most importantly mental health, substance abuse and stigma and discrimination [49]. For those having poor mental health, a mental health improvement program may also improve HIV protective behaviors. A more ‘syndemic approach’ may be needed to effectively explore how HIV risk and vulnerability are linked to other social determinants of health [49], which may differ between TGW [50, 51] and MSM; such an approach should be high on Cambodia’s research agenda.

Non-clinical interventions that may help improve the mental health situation of MSM and TGW might be the inclusion of gender variety and freedom of sexual expression in education for high school students and government officials, including those working in law enforcement and the military. Structural interventions, such as legal recognition of TGW and protective laws against discrimination and stigma of MSM and TGW may further help to increase the wellbeing of MSM and TGW in Cambodian society.

The data presented in this review suggests that there could be a significant role of crystal methamphetamine to increase sexual pleasure, or ‘chem sex’, in the HIV epidemic among sub-groups of MSM and TGW in Cambodia. The increase in crystal methamphetamine use in recent years is a prime suspect for the recent increase in HIV cases in this group. In a recent study it was also reported to be a driver of new HIV infections among MSM and TGW in Bangkok [52, 53]. However, no data was found in this review that could help design and deliver targeted HIV prevention interventions for MSM and TGW involved in the use of this drug.

In terms of HIV interventions, Cambodia has benefited from significant donor support in the past decades, such as from the President’s Emergency Program for AIDS Relief (PEPFAR) and the Global Fund to Fight AIDS, Tuberculosis and Malaria. As mentioned above, while HIV testing uptake has strongly improved since 1999, condom use among MSM and TG has been found more difficult to increase, especially with non-commercial sexual partners [54]. This phenomenon is not unique to Cambodia, but can be seen throughout Asia [54, 55] and elsewhere in the world [56]. In terms of evaluation research, the above-mentioned study was the only solid effort found in this scoping review. This is surprising if one considers that large scale randomised trials have been conducted to assess HIV intervention effectiveness among Cambodian entertainment and sex workers, for example [57].

In recent years, several studies have shown the efficacy, acceptability and feasibility of biomedical HIV preventive interventions, notably, HIV pre-exposure prophylaxis (PrEP) and antiretroviral treatment for prevention. In combination, implementation of these interventions among MSM and TGW in industrialized nations has been associated with strong reductions in new HIV infections [58–61]. It is therefore encouraging that the Royal Government of Cambodia has included PrEP as a key strategy to end AIDS by 2025 [62]. This effort started by the end of 2018 among MSM and TGW and is currently being scaled up to other populations at risk for HIV infection [62].

The recent introduction of HIV self-testing and community-based screening tests are also hopeful developments in attempts to further improve HIV testing uptake and linkages to HIV care among Cambodian MSM and TGW, as these interventions have been proven efficacious in other countries [63, 64].

It is important to keep a strong focus on HIV prevention for young MSM and TGW, where most infections can still be prevented. For older MSM and TGW, where HIV prevalence is highest, besides prevention, improving access to HIV testing and treatment are essential. For both older and younger MSM and TGW, working with online dating apps and focusing on high-risk sexual networks to improve access to PrEP will be pivotal and essential if the Cambodian HIV epidemic is to be halted.

A limitation of this paper was that not all documents reviewed were peer-reviewed; it was beyond the scope of this paper to assess the quality of these reports.

## Conclusions

While the disaggregated description of the epidemiology of HIV infection among MSM and TGW in Cambodia has improved in recent years, this scoping review found that not enough is understood about specific contexts in which sexual risk occurs, in particular the growing use of methamphetamine for sexual pleasure. Behavioral studies often do not distinguish condom use by different types of sexual behavior (i.e., anal, vaginal and oral sex) or by sexual role (i.e., insertive or receptive anal sex). Also, sex with multiple consecutive partners of different genders, changes in sexual role positioning and condom use during episodes of methamphetamine use are not well understood. Insights in these behavioral specifics are essential to target and frame more specific and tailored HIV prevention messages. While efforts have been undertaken to estimate the size of MSM population in Cambodia, the estimated number of TGW in the country remains unknown. Little information exists about Cambodian same-sex cultures and the social and cultural contexts in which HIV transmission occurs. It is also not known how effective current HIV service implementation modalities are, or how successful strategies to increase access to essential HIV prevention, testing and treatment services have been employed for MSM and TGW in Cambodia.

In order to improve HIV services for MSM and TGW and make them more effective, Cambodia will need to base the design of such services on a more comprehensive and in-depth understanding of same-sex cultures and gender-nonconforming identities, including how these differ by age, generation, class, religion, ethnicity and lifestyle. Designing more effective, appropriate, and higher quality services for a diverse audience is impossible without doing in-depth social science research on Cambodia’s multiple and increasingly diverse cultures of sexual and gender minorities.

Related to this, a better understanding is needed of which implementation modalities and approaches are most suitable to the Cambodian context. A venue-based outreach model appears to be still in place for MSM and TGW, despite many conducting much of their dating online. Guidance and training are needed for HIV service providers on how to improve their reach, appeal and messaging to attract the young, urban and often better-educated men they encounter online.

Regardless of how they are positioned and marketed, Cambodia needs to introduce and further expand access to a number of HIV intervention approaches that have proven successful in other countries, including PrEP, HIV self-testing, strategies for early identification of HIV infection and improved and better targeted HIV outreach and counseling. It is likely that a more syndemic approach to HIV, which includes addressing poor mental health, stigma and discrimination and the growing use of crystal methamphetamine and other drugs among MSM and TG, is needed in order to make HIV prevention and treatment services more equitable, acceptable and effective in the long term.

## Supporting information

S1 Checklist PRISMA scoping review checklist. (DOCX)
